# The Effectiveness and Recommendation of Motor Imagery Techniques for Rehabilitation after Anterior Cruciate Ligament Reconstruction: A Systematic Review

**DOI:** 10.3390/jcm10030428

**Published:** 2021-01-22

**Authors:** José Manuel Pastora-Bernal, María José Estebanez-Pérez, David Lucena-Anton, Francisco José García-López, Antonio Bort-Carballo, Rocío Martín-Valero

**Affiliations:** 1 Department of Physiotherapy, Faculty of Health Science, University of Granada, 18071 Granada, Spain; jmpastora@ugr.es; 2 Department of Physiotherapy, Faculty of Health Science, University of Malaga, 29071 Málaga, Spain; mariajoseestebanezperez@gmail.com (M.J.E.-P.); rovalemas@uma.es (R.M.-V.); 3 Department of Nursing and Physiotherapy, Faculty of Nursing and Physiotherapy, University of Cadiz, 11009 Cadiz, Spain; 4 University of Osuna, 41640 Seville, Spain; fjgarlop@gmail.com (F.J.G.-L.); a.antoniobc1@gmail.com (A.B.-C.)

**Keywords:** anterior cruciate ligament, motor imagery techniques, rehabilitation, physiotherapy

## Abstract

Motor imagery (MI) reported positive effects in some musculoskeletal rehabilitation processes. The main objective of this study was to analyze the effectiveness of MI interventions after anterior cruciate ligament (ACL) reconstruction. A systematic review was conducted from November 2018 to December 2019 in PubMed, Scopus, Web of Science, The Cochrane Library, and Physiotherapy Evidence Database (PEDro). The methodological quality, degree of recommendation, and levels of evidence were analyzed. A total of six studies were included. Selected studies showed unequal results (positive and negative) regarding pain, anxiety, fear of re-injury, function, and activities of daily living. Regarding the range of motion, anthropometric measurements, and quality of life, the results were not conclusive. Muscle activation, strength, knee laxity, time to remove external support, and neurobiological factors showed some favorable results. Nevertheless, the results were based on a limited number of studies, small sample sizes, and a moderate-weak degree of recommendation. In conclusion, our review showed a broader view of the current evidence, including a qualitative assessment to implement MI after ACL surgery. There was no clear evidence that MI added to physiotherapy was an effective intervention after ACL surgery, although some studies showed positive results in clinical outcomes. More adequately-powered long-term randomized controlled trials are necessary.

## 1. Introduction

Anterior cruciate ligament (ACL) injury has a high incidence in sports and recreational activities. ACL reconstruction, preoperative, and postoperative physiotherapy are evidence-based treatments; however, there is no consensus regarding the content of the rehabilitation program. Clinical guidelines for the rehabilitation of ACL show minimal variations in the physiotherapy techniques and recovery phases [1,2].

Internally, a motor act can be represented in the working memory without the realization of the external action, and this dynamic process is known as motor imagery (MI) [3]. The most current definitions already include concepts, such as sensory-perceptual processes, memory, and motor mechanisms [4,5,6,7]. MI has been suggested to be effective in reducing anxiety, tension, and pain while promoting and encouraging healing after injury, as well as improving motor performance and learning in athletes, healthy people, and people with neurological disorders [8,9,10,11,12,13]. MI is classified into two types; External (visual): The individual sees himself from the outside as if he was an actor watching his own movie. Internal (kinesthetic): The individual imagines himself performing the action; that is, he experiences sensations that may appear in real life [14]. MI projects internally a motor act when this is repeated assiduously, consciously, systematically, and with the purpose of improving performance, it is called mental practice (a term also used to define MI) [15]. Mental practice and physical movements follow the same rules and restrictions, as shown by some studies [16], so much so that it positively affects the learning of skills [9] and motor performance [10]. However, it seems that there are differences in neuronal activation, with the MI stimulus being less localized and less intense [12].

Fear and pain are the factors that most limit recovery from injuries in athletes, and these factors are quite stressful and frustrating, as well as conditioning physical performance. It would, therefore, be crucial to address these factors during rehabilitation [17,18,19]. Physiotherapy and rehabilitation research has not focused on this aspect and the influence on the recovery process [19,20]. If all these central neuromotor adaptations could be controlled or limited by health interventions, the possibility of preventing negative effects would decrease significantly [21].

Would it be possible to implement a treatment procedure that is more active during the immobilization period? Could this procedure prevent the negative effects of central reorganization, pain, and fear? Would it succeed in avoiding stress on the structures and bring about improvements in the motor control system? Would it succeed in returning the patient to his pre-injury performance level?

In ACL reconstruction, return to sports activity is an objective to be achieved, and research using MI and the hypothetic effects of interventions is justified. Therefore, the main objective of this study was to investigate the effectiveness of MI-based interventions and their degree of recommendation to be implemented in clinical practice guidelines after ACL reconstruction. As a secondary objective, we aimed to identify the different modalities of intervention within MI that can be used during the rehabilitation of the ACL, to identify changes in different variables with MI-based interventions, and to identify levels of evidence.

## 2. Materials and Methods

### 2.1. Search Strategy

This review followed the Preferred Reporting Items for Systematic Reviews and Meta-Analyses (PRISMA) guidelines [22] (PRISMA checklist in Additional file 1). The search was focused on MI and ACL, and it has been conducted in the following scientific databases: PubMed, Scopus, Web of Science (WOS), The Cochrane Library, and Physiotherapy Evidence Database (PEDro). The descriptors used in the search strategy for the databases were the following combination of words: (“mental practice” OR imagery OR “imagined movement” OR “mirror therapy” OR “movement representation techniques”) AND (knee OR “anterior cruciate ligament” OR ACL). The initial search was conducted in November 2018 and was completed with new searches updated to December 2019. The present review was registered in the PROSPERO database (registration code: CRD42020150956).

After applying the selected keywords, the entire set of documents was analyzed to identify possible duplicates between the different databases. Two authors (FJGL and ABC) independently examined the titles and abstracts of all documents. The full text was obtained if more information was requested to determine eligibility or if uncertainty prevailed between authors. Articles were evaluated in full text in order to identify those that meet the eligibility criteria. For trials published in a language other than English or Spanish, a translated version of the abstract was used to determine eligibility. Disagreements between the authors were resolved by discussion and consultation with JMPB (author), MJEP (author), DLA (author of correspondence), and RMV (author).

### 2.2. Eligibility Criteria

Eligibility criteria were based on the patient, intervention, comparison, outcome (PICO) model [23], as follows: Participants: Male and female subjects in any age category with an ACL injury to the knee, who required a reconstructive surgery; Intervention: Any intervention, synchronous or asynchronous, through the techniques included in the MI, with a minimum duration of one week (more than three training sessions); Comparison: All trials were required to have a comparison group; Outcomes: Any clinical outcome, including measurements based on pain, strength, recovery time, fear of relapse, disability, or function (physical, social, or psychological), was analyzed.

Economics and cost-effectiveness were not considered in the results, nor were patient or physician satisfaction, as well as outcomes that measure adherence or adherence to the rehabilitation program.

### 2.3. Study Selection

**Figure 1:**
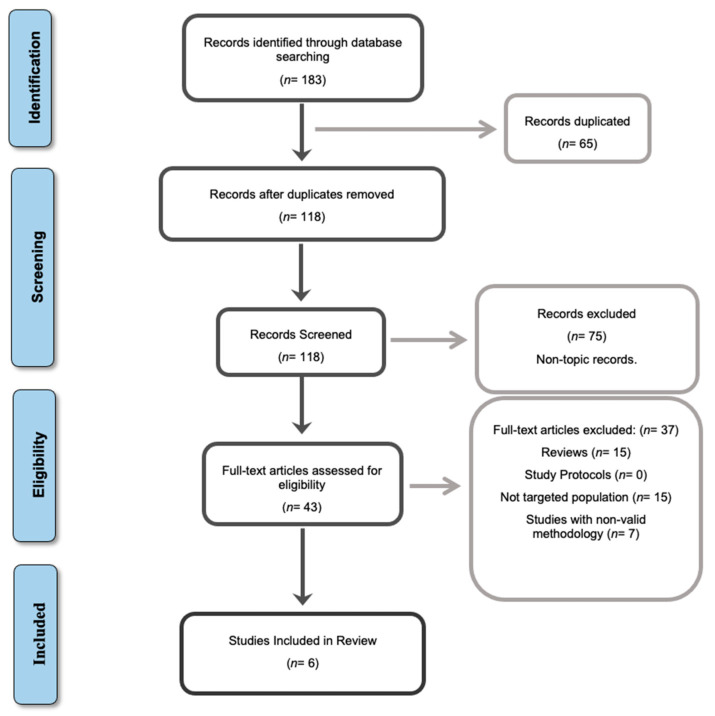
Information flowchart of the different phases of the systematic review, according to the Preferred Reporting Items for Systematic Reviews and Meta-Analyses (PRISMA) guidelines.

Randomized clinical trials (RCTs) and controlled clinical trials were considered. Articles that did not target our study population or their methodology were not valid in relation to the established criteria, as well as study reviews, case reports, pilot studies, and protocols were excluded. The main steps related to the bibliographic search phase are presented in Figure 1, using the flowchart.

### 2.4. Evaluation of Methodological Quality, Level of Evidence, and Degree of Recommendation

An important and fundamental step in conducting a systematic review is to assess the methodological quality of trials. In fact, the methodological quality of the papers provides clinicians with information on whether the results of clinical trials should influence their clinical practice [24]. Thus, the methodological quality and risk of bias were assessed using the different items recorded on the PEDro scale (Institute for Musculoskeletal Health at the University of Sydney and Sydney Local Health District, New South Wales, Australia) [24,25] based on the Delphi list [26], which is considered a useful tool for carrying out evaluation methodology in scientific research. The PEDro scale scores 10 items: specified choice criteria, random allocation, hidden allocation, group similarity at baseline, subject blinding, assessor blinding, therapist blinding, one of the key outcomes obtained from more than 85% of subjects, analysis of a key outcome with intent to treat, statistical comparisons between groups for at least one key outcome, and point and variability measures for at least one key outcome. Articles are rated present (1) or absent (0), and a score of 10 is obtained by summation [24]. The scale includes an additional criterion (eligibility criterion) to assess external validity but is not counted in the score. According to Moseley et al., studies with a PEDro score ≥ 5 are considered to have a low risk of bias and high methodological quality [27]. A study with a PEDro score ≥ 6 is considered level 1 evidence (6–8 = good, 9–10 = excellent), and a study with a score ≤ 5 is considered level 2 evidence (4–5 = acceptable, <4 = poor) [28].

Levels of evidence help us guide the search to the type of evidence most likely to provide reliable information. These have been designed to be used as an aid for doctors, researchers, or patients trying to find the best evidence [29]. In addition, the grades of recommendation describe the strength and rigors of the study. The levels of evidence and grades of recommendation were measured by the Oxford Centre for Evidence-Based Medicine (OCEBM Levels of Evidence Working Group, Oxford, United Kingdom) [30].

## 3. Results

A total of six studies were finally included. The main findings of the systematic review are presented in Table 1, including characteristics, authors, population, participants, mean age, and study design. In addition, type of intervention, duration, outcome measures, measurement instruments, and final results were included. The level of evidence and degree of recommendation based on CEBM were reported.

A subanalysis of outcomes synthesizing results is presented in Table 2. The methodological quality of included studies is shown in Table 3 based on the PEDro Scale.

### 3.1. Participant Characteristics

A total of 228 participants with ACL reconstruction received MI intervention. Great heterogeneity was found among the included studies with respect to the sample, which ranged from 10 [32] to 101 [31]. In the analysis of the population, five studies directed only at ACLs were observed; however, the remaining article by Wilczynska et al. (2015) [32] is directed at more knee pathologies, such as meniscus repair and patellar dislocation.

### 3.2. Intervention Characteristics

In all studies, a standard physiotherapy intervention was observed in both the experimental group (EG) and the control group (CG), and MI intervention was included in all EGs. On the one hand, two articles focused their intervention on visual MI [31,33] through the visualization of therapeutic videos. On the other hand, there was an article [34] that mainly focused on kinesthetic MI, favoring the imagination of different actions on the part of the subjects. Finally, three articles [20,32,35] focused their intervention on both types of MI, both visual and kinesthetic. In short, all MI interventions followed a general structure based on cognitive and motivational aspects and examples of functional tasks, both described and executed, which helps to recover. It should be noted that two studies [20,35] introduced relaxation techniques into the intervention within the EG, while another study [34] introduced relaxation, but only in the pre-intervention phase. The duration of the different interventions ranged from 4 video viewing sessions [33] to 24 sessions [31].

### 3.3. Outcomes

In the selected study, we found a significant lack of homogeneity with respect to outcomes and instruments, which did not allow us to undertake a detailed meta-analysis. Some homogeneous results were found in pain, ROM, and anxiety or fear of reinjury. The pain was the most measured outcome [20,32,33,34] in four articles; ROM in three articles [32,33,34]; anxiety or fear of reinjury in three articles [20,31,33], and function in the other three articles [31,33,34]. The rest of the outcomes are shown in Table 2 with subgroup analysis.

### 3.4. Methodological Quality, Level of Evidence, and Degree of Recommendation

The methodological quality of included studies is shown in Table 3. The studies included in the review had a total score on the PEDro scale of 5–7. Of the six studies included in the review, three articles [31,32,35] had a PEDro scores between 6 and 7 and were considered level 1 evidence (good (4/6)), and three studies [20,33,34] with a score of 5 were considered level 2 evidence (acceptable (2/6)). Based on the PEDro scale, 50% of included studies were considered as good evidence.

Regarding evidence according to CEBM, we found one study [31], with level 1 of evidence and grade A of recommendation, which is considered high quality; one study [33] with level 2 of evidence and grade B of recommendation, which is considered moderate quality, and the rest of studies [20,32,34,35] level 3 of evidence and grade C of recommendation, which is considered low quality. Following the recommendations of the CEBM, the quality of a recommendation may be adjusted down if there are limitations to study design or implementation, imprecise estimates, variability in results, indirect evidence, or presence of publication bias [29]. Reviewed studies show several issues like no sample size calculation [20,32,34,35], inability to recruit participants [35], no effect size [32], publication in no JCR journal [32], absence of blinding or low number of subjects in both groups, experimental or control group [20,32,34,35].

## 4. Discussion

Our review shows a broader view of the current evidence, including a qualitative assessment to implement the MI technique after ACL surgery.

A previous systematic review by Rodriguez et al. [18] concluded that MI techniques are effective as a psychological intervention, which could reduce post-injury consequences with positive functional and mental outcomes with scarce and no clear evidence. ACL injury has led to numerous different research techniques and therapeutic approaches and has gained an important boom in recent decades. Postoperative recovery has been mainly focused on improving the movement and strength of the knee joint, while stressful psychological factors have not been taken into account [36]. Studies have suggested that fear of re-injury may play an important role in returning to the same level of activity after surgery [20]. The application of MI techniques in the recovery process may help alleviate these factors [37]. In psychological interventions, another widely used mental process is the observation of actions. This evokes an internal process in which a person is exposed to visualize in real-time how another person performs a movement, action, or exercise. Both MI and the observation of actions are internal mental processes that manage to activate neuronal circuits. The difference resides in that the media from which the mental image is obtained is externalized; it is not purely based on our own imagination; instead, we obtain the image in an external way [38,39,40].

In this discussion section, we have talked in the first place about the contributions in the number of studies on this subject and the lack of follow-up. Secondly, the heterogeneity, generalizations, and target population are addressed. Third, results, outcomes, and methodological limitations are discussed. Fourthly, there is a review of the difficulties inherent in research with MI interventions. Finally, we have addressed future lines of research and foresight.

As for the first aspect, the lack of available studies on this subject is striking. Only 118 studies are found, as can be seen in the identification and screening phases of the flowchart. MI has not been studied in depth until now to explore its application in musculoskeletal injuries. This research includes studies from 2001 to the present day, since it is true that almost two decades have passed and not enough research has been done to reach relevant conclusions. Most of the studies included in the review are of moderate or weak methodological quality. In addition, a lack of continuity in the studies can be extracted, which justifies the fact that no follow-up period has been found. It should be pointed out that research in the ACL rehabilitation process has frequently shown results at 4, 6, and 12 months after the injury; this follow-up is not presented in any of the studies included in this review, which is a major limitation in the available evidence.

For the second aspect pointed out in the discussion, the homogeneity of the population samples is a guarantee of the quality of the results. However, in this systematic review, we find a population range ranging from 10 to 101. This is a major limitation of the study, and only two studies [31,33] are based on sample sizes, which can be considered as relevant (*n* = 101 and *n* = 58, respectively), being the rest of the results based on groups with 13, 10, 7, or 5 patients in the experimental groups, and equal or smaller control groups. It is observed that the majority of the target population are athletes with special intrinsic psychological characteristics. This leads to a lack of veracity if the results would be extrapolated to the general population. Only three articles [33,34,35] specifies the type of graft, using the Kenneth Jones technique, one-third of the central patellar tendon, autograft hamstring-tendon, and homogeneous hamstring-tendon, respectively, which can have a significant influence on clinical outcomes. We believe this is an interesting fact to highlight, as it may provide useful information for future approaches.

Regarding the third aspect of the discussion, recent systematic reviews affirm the positive effects of MI intervention, in contrast with studies highlighting the lack of evidence [41]. Our review shows a broader view of the current evidence. In the selected study, we find a significant lack of homogeneity with respect to outcomes and instruments, which makes it impossible to perform a meta-analysis, as reported in the results.

Pain has been measured in four studies [20,32,33,34], showing results in favor of the experimental group in three of them, as seen in Table 3. However, there are differences in the evaluation instruments, in the scales, and in the proper definition of the outcome (expected pain/current pain), which makes it difficult to reach and unify conclusions. Knee strength shows positive results in only one of the studies; ROM and anthropometry do not show positive results, which is expected based on the type of intervention without the functional activity of the locomotive system.

This review has found only three studies measuring anxiety and fear of re-injury [20,31,33], with discrepancies between the positive results and the improvement of this outcome between the studies. These variables should be considered as principals in the research or at least considered as a strange or confusing variable due to their high impact on the final results of the recovery process. Furthermore, it is interesting to mention that only one study [35] incorporates neurobiological factors, such as dopamine and noradrenaline, as they are hormones related to motor function and have an influence on learning and motor performance. Other variables have been measured, providing results but, in general, are not consistent, or the impact in the evidence is limited.

With respect to the number and duration of sessions, there is no clear pattern, so we believe that this may limit the quality of the results. In fact, the evidence recommends treatments limited to 20 min for healthy people, being evident a negative relationship between the duration of the practice and the effect produced. For this reason, it is important to highlight how much time of the intervention will be devoted to the preparation of the patient for MI treatment [42,43,44]. All the included studies incorporate standard physiotherapy in conjunction with MI for the experimental group, although not all specify which standard physiotherapy procedures are used. In addition, it is important to know both MI techniques used, such as visual and kinesthetic, as well as to mention that there is no clear criterion of choice between the two. The study by Lebon et al. [34], which uses only Kinesthetic MI, argued that the use of this technique increases the probability of providing better biofeedback of joints and muscles, as well as increasing motor excitability, mainly at the supraspinal level [45,46]. However, two other studies use mainly visual MI [31,33], while the remaining studies use both types. In all of them, positive results are seen, so that statement must be questioned.

Which technique is more effective? Do they depend on the intervention or the number of times it is performed? The answer to these questions is currently unknown. Finally, there are studies that use relaxation techniques prior to MI treatment and others that use them during the treatment session. This information is relevant as we know that relaxation is not essential for MI treatment and may even limit its benefits when the end result sought is to improve learning and motor performance. In other words, previously used can be useful to increase concentration in the task; however, during the session can reduce the corticomotor activity being this contrary to what is pursued [47,48].

Fourthly, we have highlighted the difficulties inherent in carrying out studies with this type of intervention. During the search, validated clinical results have been observed, but small samples are used in the studies. In addition, differences in interventions, treatment period, and follow-up create doubts about whether the techniques offer comparable results or not. Another common problem in MI studies is the lack of blinding of therapists and patients. There is evidence that in clinical trials in which the assignment of evaluators is not hidden, therapists and participants present lower quality methodology with respect to blinded procedures [49].

How might this issue be addressed in future research? What future lines of research are left open? Modifications in several methodological aspects are fundamental for future research. MI should be conceptualized, coded, classified, and grouped in a similar way to the physiotherapy protocols, which will allow detailed identification of when the effect is due to the type of intervention. The frequency of MI for the experimental group should be the same as for the control group, thus avoiding biases related to the intervention. Greater homogeneity is needed, especially in the sample size, as well as in terms of the type, duration, and follow-up of the intervention. Studies that show negative results should be published, avoiding publication bias. Improving the quality of studies (blinding) should be addressed. Studies of high methodological quality with a long-term follow-up period and conclusive results should be conducted to confirm that MI proves to be clinically relevant in treatment after ACL reconstruction. MI appears as another therapeutic option, whose benefits could be valid after discharge from hospital in patients suffering from ACL injuries and could improve health interventions and physiotherapy clinical practice. Future challenges include identifying whether positive outcomes are due to the type of intervention or the increased frequency and intensity that MI allows. At present, the evidence and arguments that revealed the profound conceptual implications, and the empirical problems in the theory of MI, have not yet been solved.

It is interesting to note the limited evidence regarding pre-surgical programs with MI prior to ACL reconstruction, as the vast majority of trials focus on the postoperative period. Therefore, how it could benefit and what would be the effectiveness of MI if it is started to be used from the moment of injury, another prospective question to solve. There are still small databases for MI and ACL studies, although these may provide useful data on clinical outcomes and future lines of research; in fact, most studies conducted on the knee address knee replacement rather than ACL injury. Our findings are aligned with the most recent reviews regarding the need for future research, concluding that stronger and more robust studies are needed.

One of the biases identified is that MI groups have more frequent contact with health professionals, so they are likely to receive additional assistance. Consequently, this could create a bias if positive outcomes were not dependent on the method of intervention the patient perceives, which is receiving extra care.

## 5. Conclusions

To answer the main objective: Our research showed that there was no clear evidence that MI added to physiotherapy was an effective intervention in ACL after surgery. The included studies showed unequal results (positive and negative) regarding pain, anxiety, fear of re-injury, function, and activities of daily living. In terms of the range of motion, anthropometric measurements, and quality of life, the results were not conclusive. Muscle activation, strength, knee laxity, time to remove external support, and neurobiological factors showed some favorable results. Nevertheless, these results were based on a limited number of studies with small sample sizes. Regarding the quality of the evidence, we judged this to be a moderate-weak recommendation.

To meet secondary objectives: the studies included in this review offered different motor imaging interventions (motor and kinesthetic), which were used individually and simultaneously, providing unequal results on different outcomes.

More adequately powered long-term randomized controlled trials are necessary. Additional evidence is needed to evaluate whether MI combined with physiotherapy is efficacious in people after ACL surgery. Our research added data to previous knowledge, including a qualitative assessment on MI trials, and showed a broader view of current evidence.

## Figures and Tables

**Table 1:** Main results.

Study	Condition	Participants	Average Age	Methodological Quality	Intervention	Duration	Outcomes	Results
Cupal et al. (2001) [20] RCT	ACL Reconstruction	N: 30 EG: 10 CG: 10 PG: 10	28.2 (8.2); 14/16	8/3/C	EG: Relaxation and MI (visual, kinesthetic, and motivational imagery) + standard physiotherapy. CG: Standard physiotherapy. PG: Attention, encouragement, and support.	10 sessions every 2 weeks with a duration of 10–15 min each session. Measurements at two weeks (basal) and six months after surgery.	1. Anxiety of re-injury by questionnaire 11-point scale—ranging from 0 (no concern) to 10 (extreme concern). 2. Perception of pain by scale ranging from 0 (no pain) to 10 (extreme pain). 3. Knee strength through Cybex 6000 Isokinetic dynamometer.	There was a significant improvement in knee strength in the EG (0.83) compared to the PG (0.63) and CG (0.66); re-injury anxiety was significantly lower in EG (1.10) compared to both PG (4.00) and CG (3.40) groups. Pain also showed decrease in the EG (0.70) compared to both GP (2.70) and CG (2.70) groups (*p* < 0.05) 24 weeks post-surgery in EG.
Zaffagnini et al. (2013) [31] RCT	ACL reconstruction	N: 101 EG: 51 CG: 50	33 (11.1); 21/80	9/1/A	EG: Standard physiotherapy + therapeutic vision video. GG: Standard physical therapy + video of unfavorable information.	3 times a week for 2 months. Preoperative (basal) measurement and three months after surgery.	1. Health-related quality of life through health questionnaire (SF-36). 2. Knee function by means of subjective and objective IKDC questionnaire. 3. Fear of re-injury by means of TSK. 4. Level of activity through Tegner. 5. Time to remove external assistance.	There were no significant differences in health-related quality of life between groups. In knee function, significant improvement in subjective IKDC was observed in the EG (82.0) compared to the CG (71.0) (*p* < 0.05). Fear of re-injury greater improvement in the EG (28.1) was observed compared to the CG (32) (*p* < 0.05). There was a significant improvement in time to remove external support in the EG (20.9) compared to the CG (26.5) (*p* < 0.05).
Wilczynska et al.(2015) [32] RCT	Knee arthroscopy (replacement meniscus, ACL reconstruction (5), patellar dislocation).	N: 10 EG: 5 CG: 5	35	6/3/C	EG: Kinesthetic and motivational MI (visualization techniques of functional recovery and total efficiency) + standard physiotherapy. CG: Standard physiotherapy.	15 sessions.	1. Circumference of the operated and non-operated limb (6 to 10 cm from the patella base). 2. Scale of pain by LPS. 3. ROM in flexion of the operated and non-operated leg.	Both groups showed a significant reduction in pain; however, the EG reported less pain after surgery regardless of time compared to the CG. Similar results were found for ROM and leg circumference operated on “ROM day 1 (79 vs. 83) to day 15 (118 vs. 119)”. “Leg circumference day 1 (417.5 vs. 419.9) and day 15 (417.2 vs. 425.5)”.
Maddison et al. 2006) [33] RCT	ACL Reconstruction	N: 58 EG:30 CG:28	30 (ND) 19/39	5/2/B	EG: Standard physiotherapy + video display (edited interviews + real examples of functional tasks) in the preoperative, before discharge, two and six weeks postoperative. CG: Standard physiotherapy.	From baseline (preop) to six weeks postop. Two videos of 9 and 7 min, displayed twice each.	1. Perception of pain expected by scale between 0 (no pain) and 100 (max. pain). 2. Anxiety by means of State-Trait Anxiety Inventory (STAI). 3. Self-efficacy through self-efficacy scales: (CSE), (WSE), and (ESE). 4. Clinical evaluation of the knee using the IKDC standard subjective and objective assessment form. 5. ROM by goniometry.	Significant differences (*p* < 0.05) were found in support of the EG for the expected pain. For both groups, no significant effect was found for actual pain and for anxiety. Greater self-efficacy for crutches, walking, and exercise was observed in the EG compared to the CG only at the time before loading without crutches. The objective IKDC score improved in favor of the EG (*p* < 0.05). The subjective IKDC scores approached significance, with the EG obtaining higher scores (less disability) at 6 weeks. There was no difference for ROM.
Lebon et al. 2011 [34] RCT	ACL reconstruction	N: 12 EG: 7 CG: 5	28.5 ± 5.0 (UD) 2/10	5/3/C	EG: Rehabilitation with kinesthetic MI + standard physiotherapy. CG: Cognitive neutral task + standard physical therapy.Relaxation was performed prior to the beginning of the intervention.	12 sessions in 28–34 days/15 min of the session every two days.	1. Isometric activation of the medial vasculature by EMG during maximum knee extension. 2. Pain by means of the visual analog scale. 3. Ability to perform DLA with lower extremity injury using the Lower Extremity Functional Scale (LEFS). 4. Magnitude of surgical effusion and atrophy by the circumference of the knee above the kneecap and circumference of the thigh 15 cm above the kneecap. 5. ROM of the knee with a goniometer.	The EMG pretest activity showed similar results in both groups. In contrast, the post-test EMG activity showed an increase of muscle activity in both groups, being significantly higher in the MI group (85.36% vs. 51.56% compared to the healthy leg) (*p* < 0.05). Pain decreased for both groups, with no significant difference between them. The ability to perform DLA and anthropometric measurements showed no significant difference between the two groups. Anthropometric measurements.
Madisson et al. (2012) [35] RCT	ACL reconstruction	N: 21 EG: 13 CG: 8	34.86 (8.84); 8/13	7/3/C	EG: MI (visual and kinesthesia) + relaxation + standard physiotherapy. CG: Standard physiotherapy.	9 sessions in total. Preoperative (basal) measurements and two, six, and twelve weeks after surgery.	1. Knee strength using Cybex 6000 Isokinetic dynamometer. 2. Knee laxity using KT1000 Arthrometer. 3. Neurobiological factors through a 24-h urine sample. 4. Self-efficacy by questionnaire—Athletic Injury Self Efficacy (AIESQ). 5. Atlletic Injury Imagery Questionnaire (AIIQ-2; rehabilitation images).	Knee laxity was significantly lower in the EG (5.25 mm to 15 mm) compared to the CG (3.73 mm to 50 mm) after 6 months. Urine samples reflected significantly lower levels of noradrenaline and dopamine at 2, 6, and 12 weeks in the EG. The use of imaging was advantageous to the EG. There were no statistical differences for knee extension strength.

DLA: Daily life activities; CSE: Self-Efficiency Scale with Crutches; RCT: Randomized controlled trial; EMG: Electromyography; ESE: Self-Efficiency Rehabilitation Exercises Scales; CG: Control group; EG: Experimental group; IKDC: International Knee Documentation Committee; PG: Placebo group; MI: Motor imagery; ACL: Anterior cruciate ligament; LPS: Pain Scale Laitinen; N: Nº patients; ROM: Range of movement; TSK: Kinesiophobia Scale of Tampa; WSE: Self-Efficiency Walking Scale.

**Table 2:** Outcomes subgroup analysis.

Outcomes	Cupal et al. (2001) [20] RCT	Maddison et al. 2006) [33] RCT	Lebon et al. 2011 [34] RCT	Madisson et al. (2012) [35] RCT	Zaffagnini et al. (2013) [31] RCT	Wilczynska et al. (2015) [32] RCT
Perception of actual pain	+	(−)	(−)			+
Expected pain		+				
Knee strength	+			+		
Anxiety	+	(−)	(−)			
Fear of re-injury	+				(−)	
Function, self-efficacy, DLA		+	(−)		+	
ROM		(−)	(−)			(−)
Muscle activation EMG			+			
Anthropometry			(−)			(−)
Knee laxity				+		
Quality of life						(−)
Neurobiological factors				+		
Time to remove external support						+

RCT: Randomized controlled trial; DLA: Daily life activities; EMG: Electromyography; ROM: Range of movement; **+** (Positive effect or significant difference in favor of experimental versus control group); (−) No difference or no improvement between groups.

**Table 3:** Methodological quality according to the PEDro scale.

Criteria	Cupal et al. [20]	Madisson et al. [33]	Lebon et al. [34]	Maddison et al. [35]	Zaffagnini et al. [31]	Wilczynska et al. (2015) [32]
Eligibility criteria	N	Y	N	Y	N	Y
Randomization	Y	Y	Y	Y	Y	Y
Allocation concealed	N	N	N	Y	Y	N
Baseline comparability	Y	Y	Y	Y	Y	Y
Subject blinding	N	N	N	N	N	N
Therapist blinding	N	N	N	N	N	N
Evaluator blinding	N	N	Y	Y	Y	N
Appropriate continuation	Y	N	N	N	Y	Y
Intention to treat	N	Y	N	Y	N	Y
Comparison between groups	Y	Y	Y	Y	Y	Y
Specific measurements and variability	Y	Y	Y	Y	Y	Y
Total PEDro Score	5	5	5	7	7	6

PEDro: Physiotherapy Evidence Database. The eligibility criteria do not contribute to the total score. Y: Yes; N: No.

## Data Availability

No new data were created in this study. Data sharing is not applicable to this article.
